# Tuning three-dimensional nano-assembly in the mesoscale via bis(imino)pyridine molecular functionalization

**DOI:** 10.1038/s41598-022-04851-6

**Published:** 2022-01-17

**Authors:** Ryan Brisbin, Mark Bartolo, Michael Leville, Arya K. Rajan, Basharat Jahan, Kara E. McCloskey, Ajay Gopinathan, Sayantani Ghosh, Ryan Baxter

**Affiliations:** 1 Department of Chemistry and Biochemistry, University of California, Merced, CA 95343 USA; 2 Material and Biomaterial Sciences and Engineering, School of Engineering, University of California, Merced, CA 95343 USA; 3 Department of Physics, University of California, Merced, CA 95343 USA

**Keywords:** Organic chemistry, Chemical synthesis, Condensed-matter physics, Information theory and computation, Nanoscale materials

## Abstract

We investigate the effect of bis(imino)pyridine (BIP) ligands in guiding self-assembly of semiconducting CdSe/ZnS quantum dots (QDs) into three-dimensional multi-layered shells with diameters spanning the entire mesoscopic range, from 200 nm to 2 μm. The assembly process is directed by guest–host interactions between the BIP ligands and a thermotropic liquid crystal (LC), with the latter’s phase transition driving the process. Characterization of the shell structures, through scanning electron microscopy and dynamic light scattering, demonstrates that the average shell diameter depends on the BIP structure, and that changing one functional group in the chemical scaffold allows systematic tuning of shell sizes across the entire range. Differential scanning calorimetry confirms a relationship between shell sizes and the thermodynamic perturbation of the BIP molecules to the LC phase transition temperature, allowing analytical modeling of shell assembly energetics. This novel mechanism to controllably tune shell sizes over the entire mesoscale via one standard protocol is a significant development for research on in situ cargo/drug delivery platforms using nano-assembled structures.

## Introduction

Advances in synthesis and assembly of nano-scale materials have offered routes towards meeting the increasing demands of precision engineering on progressively smaller length scales^1,2^. Many critical technologies, including computer circuitry, and medical and energy devices rely on specialized nano-scale materials for optimum performance. Strategies for assembling meso-scale constructs from nanoparticles (NPs) generally fall into two categories: ‘top down’ or ‘bottom up’. The former offers high precision and spatial order^3,4^, leading to the fabrication of ‘superlattices’ (SLs), but these are geometrically constrained and not easily scalable. ‘Bottom-up’ methods are driven by colloidal techniques, and SLs developed to date, including some on the microscale, are three-dimensional and compositionally heterogeneous, comprising up to three different types of NPs as building blocks^5–12^.

An intriguing subset of bottom-up self-assembly techniques is one guided by host–guest interactions of NPs (guests) with suitable functionalized surfaces dispersed in solvents (hosts)^13–18^. The resultant assemblies are usually amorphous, possessing short-range order. However, the potential for modularity leading to structurally diverse and non-planar assemblies represents distinct advantages over SLs^19–21^. The host, when exposed to an appropriate set of conditions, directs the NPs to assemble into complex structures. The most common host materials are polymers and liquid crystals (LCs), and the assembly is driven by a phase transition, where the NPs are segregated at interfaces between the phases, leading to the formation of the superstructures. We have pioneered an LC-driven nano-assembly process^22^, where the NPs were functionalized with mesogenic (LC-like) ligands and dispersed in 4-Cyano-4′-pentylbiphenyl, a common LC, known as 5CB. As the LC host is cooled, it transitions from an isotropic (disordered) phase to a nematic (ordered) phase. During the transition, the dispersed NPs are pushed together at the phase boundary, where they form three-dimensional shell structures, with walls consisting of multiple layers of NPs. We further demonstrated that these nano-assembled shells were capable of encapsulating and retaining cargo over a period of months^13^. These structures showed tremendous potential for cargo delivery in biomedical and bioengineering applications^23–25^, as they could be optically activated to rupture within seconds by photothermally disrupting the shell integrity. This approach, though a novel and versatile form of nano-assembly, has practical limitations. One is that the mesogenic ligand used in driving the assembly are highly cytotoxic; the other is the lack of control in tuning shell sizes, leading to structures too large to be suitable for in vitro applications^26,27^. In this current work, we move away from mesogenic ligands and focus on the bis(imino)pyridine (BIP) family of ligands^28^ which, in addition to being biocompatible, allow structural modularity and offer a route to fundamentally understand and thereby manipulate the assembly process to controllably tune the shell sizes. Using CdSe/ZnS core/shell quantum dots (QDs) functionalized with BIP molecules and dispersed in 5CB, we demonstrate that subtle changes to the BIP structure results in a tunable assembly process, yielding shells of predictable sizes. Dimethyl BIP, unsubstituted BIP and diisopropyl BIP create shells with average diameters of 200 nm, 400 nm and 900 nm, respectively, confirmed using scanning electron microscopy (SEM) and dynamic light scattering (DLS). We further determine both the essential chemical structure needed for the assembly process using a series of control ligands, and the thermodynamic parameters associated with the isotropic-nematic phase transition of 5CB that allows size tunability. Data from differential scanning calorimetry (DSC) of LC-BIP mixtures agree with our analytical model of how shell radius is predicated by the free energy balance of BIP-functionalized QDs in the host at the interface of the two. Finally, cell toxicity studies confirm that the dimethyl- and isopropyl-BIP ligands are non-toxic even at high concentrations, thereby making practical applications for controlled delivery of growth factors into developing tissues a possibility.

## Shell assembly

The shell assembly process begins with QD surface functionalization. Octadecylamine (ODA)-capped CdSe/ZnS QDs (NN-Labs) with a core diameter of 4.8 nm (5–10% size inhomogeneity) undergo ligand exchange under inert conditions. The QDs are added to a hexane solution containing an excess of the modifying ligands relative to the native ODA. This solution is incubated for 5 min, and then purified with acetonitrile and chloroform. The QDs are then separated by centrifugation and redispersed in toluene. Following surface modification, a 1.5 mL microcentrifuge tube (mct) is filled with 20 µL of 5CB (Sigma Aldrich) in isotropic phase and mixed with 20 µL of functionalized QDs diluted to 0.5 mg/mL in toluene. The QD-LC mixture is heated to 50 °C in a sonication bath for a minimum of 5 h to obtain complete QD dispersion within the isotropic LC. The mct is then cooled to 25 °C, during which, as the LC is driven through its isotropic-nematic transition temperature of 34.4 °C, the QDs are expelled from the ordered regions to minimize free energy and as they are confined in shrinking volumes within the host, π–π interactions between the ligands drive self-assembly to form shells.

**Figure 1:**
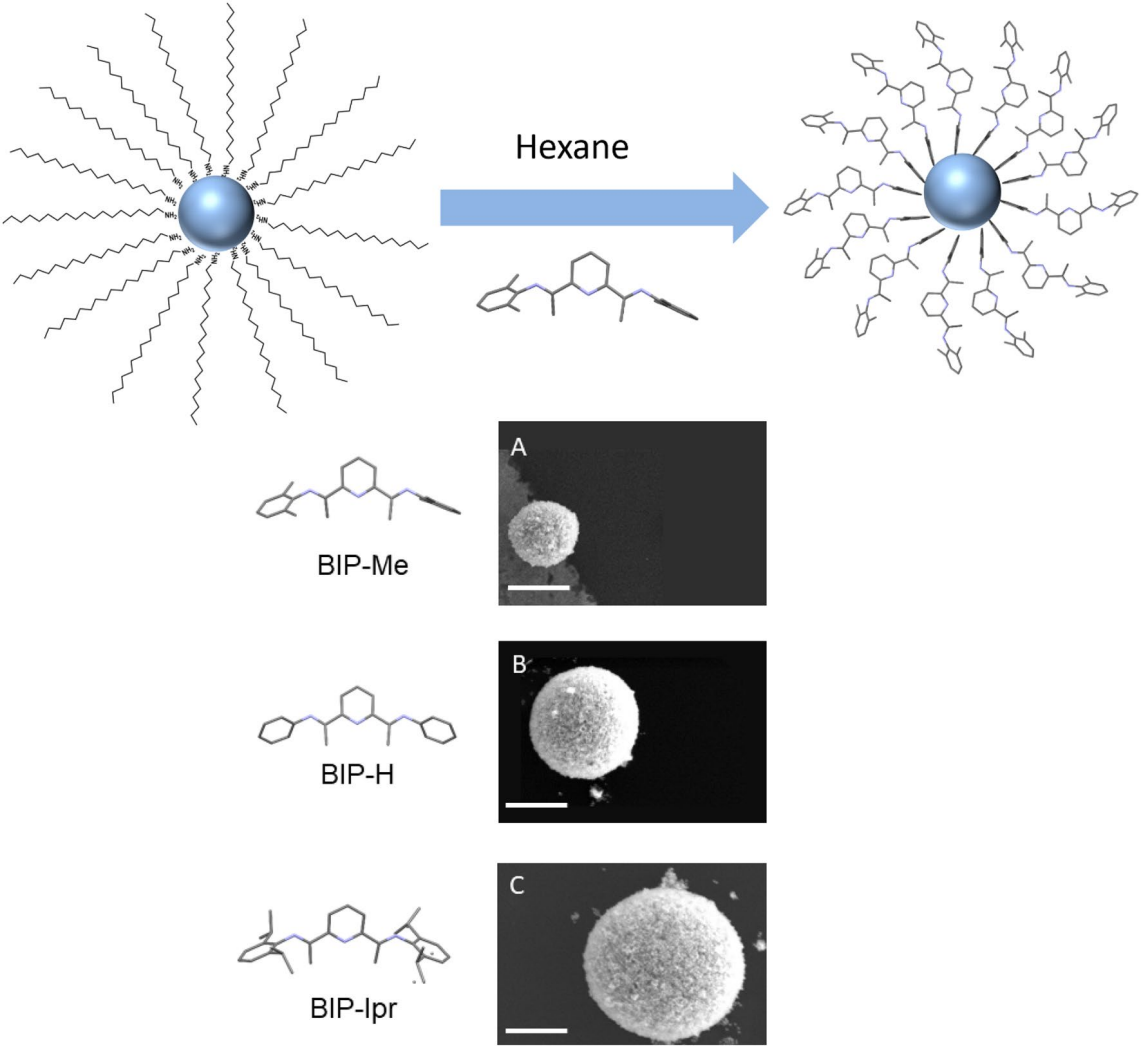
Top: Cartoon depicting ligand exchange at QD surface. Bottom (**A**–**C**): SEM images of QD shells formed using BIP-Me, BIP-H and BIP-IPr, respectively. Ligand structures are shown to the left. All scale bars are 200 nm.

**Figure 2:**
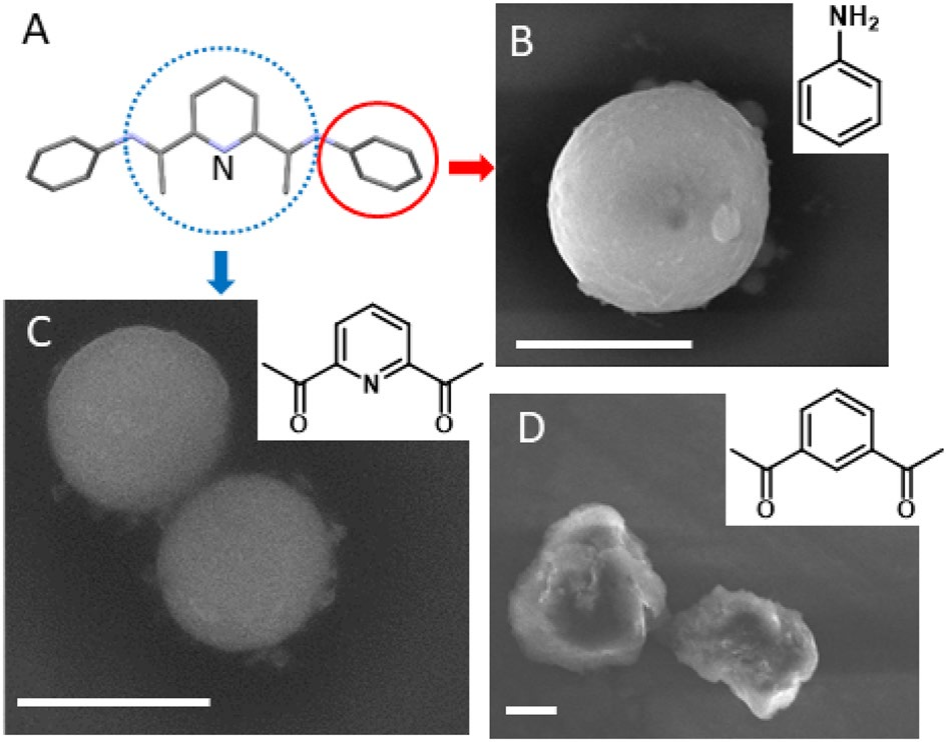
(**A**) Schematic outlining the components of BIP used as control. Dashed and solid circles highlight the aromatic/nonaromatic lewis basic nitrogens (DAP/aniline). SEM images of QD shells formed using (**B**) Aniline, (**C**) 2,6-DAP and (**D**) 1,3-DAB. All scale bars are 1 µm. Chemical structures of the component molecules are shown as accompanying insets.

Three variations of BIP ligands are examined: dimethyl-BIP (BIP-Me), unsubstituted-BIP (BIP-H) and diisopropyl-BIP (BIP-IPr). The results, following the ligand exchange and shell assembly protocols, are summarized in Fig. 1. Figure 1A–C are SEM images of shells assembled with BIP-Me, BIP-H and BIP-IPr, respectively, and show clear variation in shell diameter. Before delving deeper into the physiochemical relationships that govern shell sizes, we isolated the structural components of the BIPs, as shown in Fig. 2A, and attempted shell formation with aniline, 2,6-DAP (diacetylpyridine) and 1,3-DAB (diacetyl benzene). Figure 2B,C display the corresponding SEM images of shells formed by QDs functionalized with 2,6-DAP and 1,3-DAB, respectively. We find that QDs functionalized with aniline (Fig. 2B) and 2,6-DAP (Fig. 2C) do successfully form shells, with diameters in the range 800 nm–2 μm. However, QDs functionalized with 1,3-DAB (Fig. 2D) do not form shells, instead forming large (> 2 µm) and irregular clusters. Such clusters are typically observed when QD surfaces are functionalized with long organic chains, such as ODA. This set of results demonstrates that in addition to aromaticity, the presence of nitrogen is critical to shell formation. The requirement for aromaticity may be understood by the presence of π–π interactions between ligands as they adhere to the QD surface, but the position of the pyridine nitrogen within the ligand structure likely precludes direct atomic binding to the QDs. Alternatively, the dipole created by the presence of the pyridine nitrogen atom may enhance π–π interactions between individual ligands and between ligands and the QD surface.

**Figure 3:**
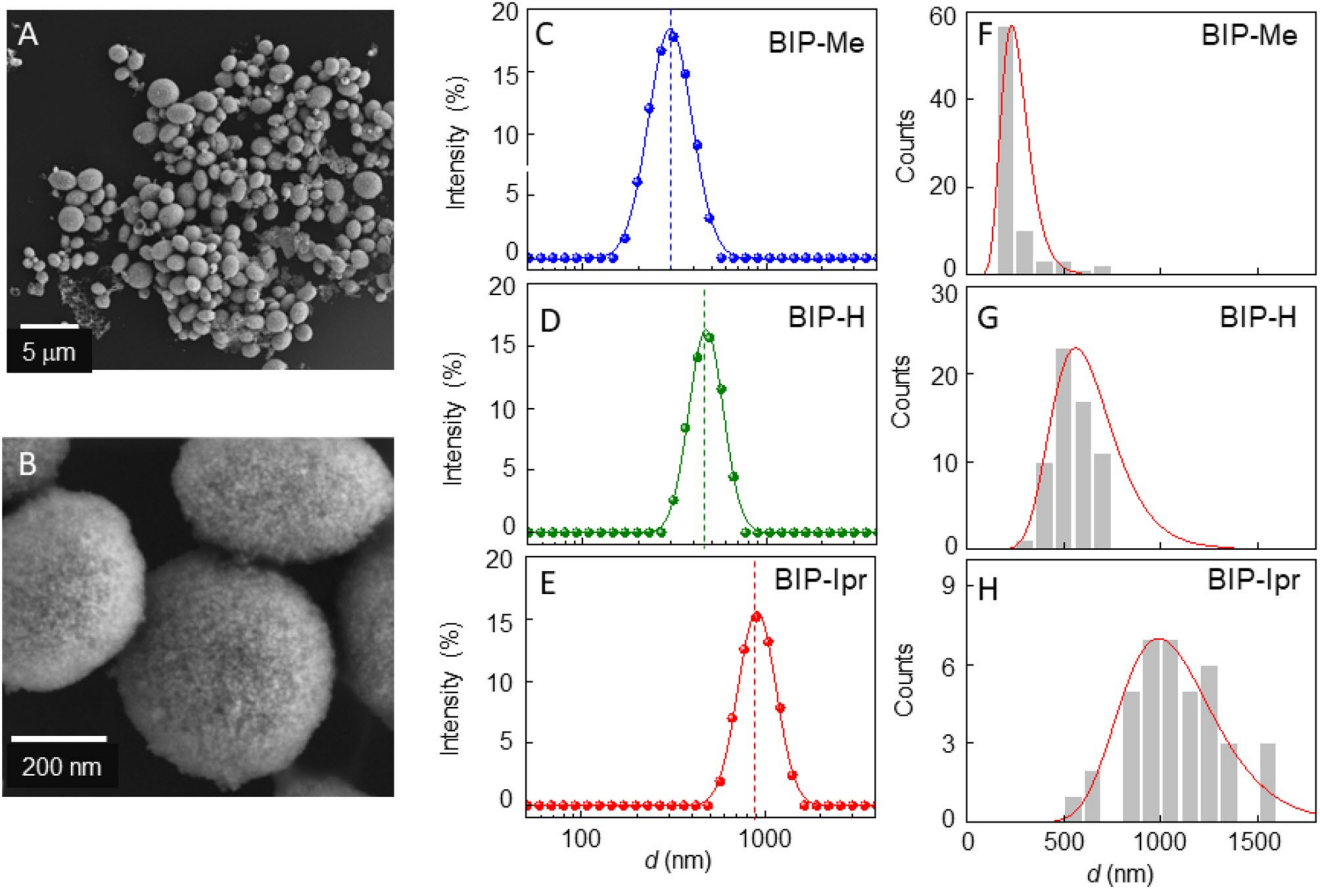
(**A**,**B**) SEM images of shell ensembles in a typical synthesis (**C**–**E**) DLS data of ensembles of shells for each of the BIP ligands. (**F**–**H**) Shell size distribution obtained from image analysis of multiple samples of each kind for the three different BIP ligands.

In Fig. 3 we turn to accurately recording the size inhomogeneity of the shells, inherent in any colloidally processed ensemble. Figure 3A,B are SEM images of shells produced in one synthesis process using BIP-IPr functionalized QDs, and clearly demonstrate significant size variation. We characterize this inhomogeneity in two ways. First, using dynamic light scattering (DLS) measurements, where samples of each type of BIP-QD shells are mixed with 40 µL of chloroform (CHCl_3_) and centrifuged for 10 min at 6000 rpm. The aliquot is removed, redispersed in CHCl_3_ for a second centrifugation, and redispersed in 250 µL of toluene by sonication. A Malvern Zetasizer Pro is used to conduct DLS at 40 °C, and the results are shown in Fig. 3C–E. These confirm the trends observed in SEM images of single shells, i.e., BIP-Me functionalized QDs form the smallest shells, with a mean diameter of 300 nm and a variance of 100 nm. For BIP-H and BIP-IPr, the mean shell diameters (variances) are 460 nm (125 nm) and 900 nm (300 nm), respectively. To obtain a better estimate of shell sizes and distributions, we additionally performed a more thorough global statistical analysis of the BIP-functionalized shells via image processing of SEM data of all synthesis attempts, a total of 232. The results are plotted in Fig. 3F–H and the results further substantiate the prior observations. However, while the correlation between BIP variants and shell sizes is reassuring, investigating the mechanism by which the sizes are tuned is of greater fundamental importance, as that would provide the means for establishing this assembly technique as a standard protocol capable of rational design.

**Figure 4:**
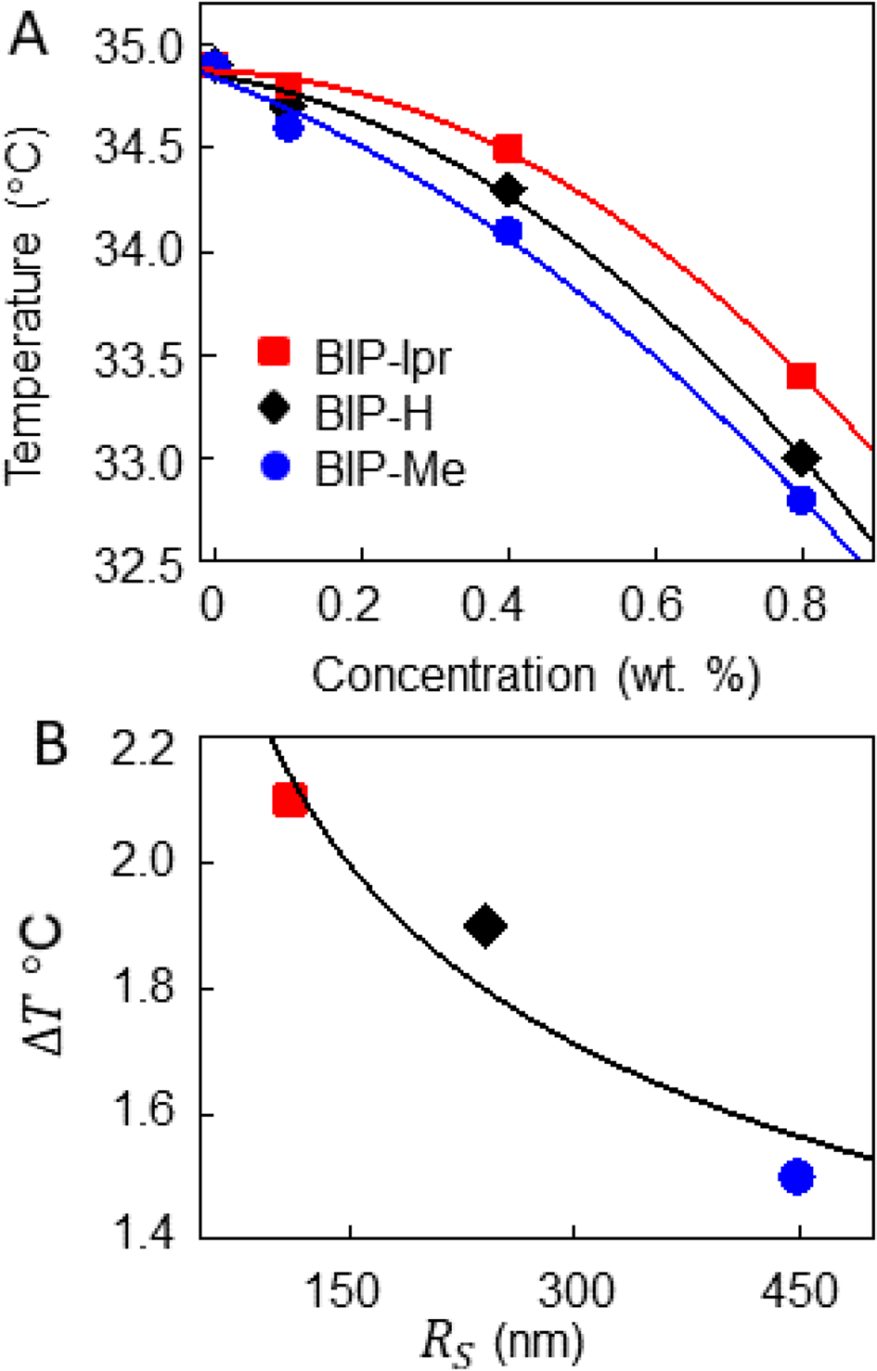
(**A**) DSC results summarized for the three BIP ligands, plotting the 5CB isotropic-nematic transition temperature with increasing ligand concentration. (**B**) Average shell radius *R*_*S*_ varying with the change in the transition temperature $\Delta T$ for each ligand. The fit is explained in the text.

**Figure 5:**
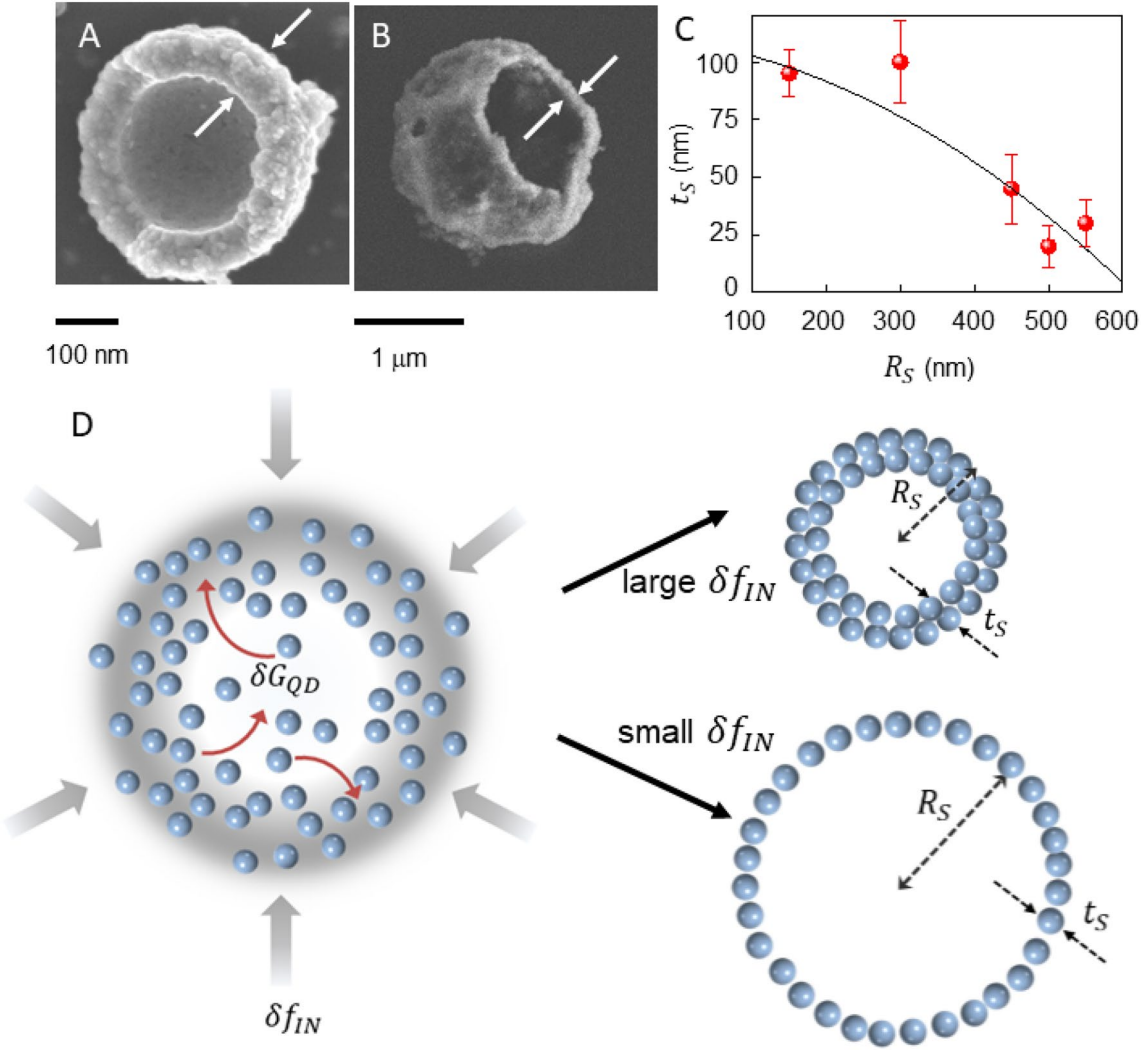
Ruptured shells of (**A**) BIP-Me functionalized and (**B**) BIP-IPr functionalized QDs showing different wall thicknesses. (**C**) Plot of *t*_*S*_ as function of *R*_*S*_. (**D**) Schematic depicting the formation. *R*_*S*_: shell radius; *t*_*S*_: shell wall thickness; *P*_*IN*_: Pressure from nematic phase boundary; $\delta f_{IN}$: free energy density change during transition.

First, we sought to ascertain whether the steric ligand parameters affect the mean shell size. Unit cell structures of the BIP ligands, obtained from crystallization data^28^, reveal that BIP-H has the smallest volume, while BIP-IPr the largest, which does not correlate to shell size, since BIP-Me functionalized QDs lead to the smallest shells. Packing density of QDs in the shell walls was next suspected to determine shell size, but with BIP-IPr QDs having the smallest inter-dot separation, this physical property was also not in agreement with observed shell size variation^28^. This lack of a relationship to any physiochemical property (lattice dimensions, torsional angles, sterics, lattice volumes) prompted an investigation of the thermodynamic effect of the ligands on the host 5CB using differential scanning calorimetry (DSC) in Fig. 4. The isotropic-nematic phase transition in LCs is weakly first order, and in scanning calorimetry measurements demonstrate peaks at the transition temperatures^29^. For 5CB alone, we observe the transition at 34.8 °C. When the measurements are repeated with the ligands added to it, the transition temperature decreases to different extents for the three variants, shown in Fig. 4A. Incorporation of additives lead to suppression of the LC transition temperature as the added materials act like an impurity^30–32^. Therefore, the increased suppression with increasing ligand concentration is logical. When the radius of the shell *R*_*S*_ is plotted against the change in transition temperature $\Delta T$ in Fig. 4B we notice a monotonically decreasing relation. A simple power law fit of $\Delta T \propto R_{S}^{ - \alpha }$ returns $\alpha = 0.22$. In addition, we can also assess the wall thickness of ruptured shells using SEM imaging. Figure 5A,B show a BIP-Me shell with a wall thickness *t*_*S*_ of 114 nm, and a BIP-iPr shell of *t*_*S*_ 29 nm, respectively. Correlating thickness *t*_*S*_ to shell radius *R*_*S*_ (Fig. 5C) seems to suggest shell walls get thinner with increasing shell diameter. Given the small population of shells that rupture, the error bars in this plot are significant and we therefore do not draw any quantitative conclusion from it.

To understand why the small changes to BIP scaffolds result in such significant variation in shell size, we develop a simple model of the assembly mechanism, shown in Fig. 5D. During the shell formation process, the shell wall is a boundary between the nematic (*N*) phase outside and the isotropic phase (*I*) inside. This boundary, or front, has an effective pressure *P*_*IN*_ associated with it that serves as the initial driving force, which in turn varies linearly^33^ with the free energy change $\delta f_{IN}$ between the two homogenous phases, leading to:

1 $$ P_{IN} = \delta f_{IN} . $$

The effective pressure additionally creates a compressive stress $\gamma$ in the shell, which can be expressed as:

2 $$ \gamma = R_{S} P_{IN} /2, $$

where *R*_*S*_ is the shell radius. As shell formation progresses, the insertion of each additional QD to the shell wall has an energy cost $\delta G_{P}$ associated with it, which can be expressed as:

3 $$ \delta G_{P} t_{S} = \gamma , $$

where $t_{S}$ is the shell thickness. Combining these equations, the shell radius is:

4 $$ R_{S} = 2t_{S} \left( {{\raise0.7ex\hbox{${\delta G_{P} }$} \!\mathord{\left/ {\vphantom {{\delta G_{P} } {\delta f_{IN} }}}\right.\kern-\nulldelimiterspace} \!\lower0.7ex\hbox{${\delta f_{IN} }$}}} \right). $$

The energy cost $\delta G_{P}$ is dominated by the QD properties, rather than the ligands, but $\delta f_{IN}$, defined as the change in free energy between the *N* and *I* phases, is directly proportional to *T*_*IN*_, the thermotropic transition temperature^34^. Therefore, the lower the value of *T*_*IN*_ (the larger the change $\Delta T$) the larger the value of *R*_*S*_ where the shell structure will stabilize. The inverse relation of *R*_*S*_ and $\Delta T$ in Fig. 4B would suggest our data agrees with this, but Eq. (4) contains the term *t*_*S*_, which, as Fig. 5A–C shows, is not constant with shell size. A logical assumption would allow us the following framework: when the phase transition begins, the isolated QDs are dispersed in the LC and the thermodynamic effect of the ligands is minimal. Therefore, the number of QDs swept into the initial bubble is independent of the BIP variant, which would imply $R_{S}^{2} t_{S} \approx$ constant. This implies $t_{S} \propto R_{S}^{ - 2}$ and incorporating this in Eq. (4) implies $\delta f_{IN} \sim \Delta T \propto R_{S}^{ - 1/3}$, close to the 0.22 obtained. The discrepancy could be attributed to the fact that we have assumed $\delta G_{QD}$ is unchanged with *R*_*S*_.

## Cytotoxicity studies

**Figure 6:**
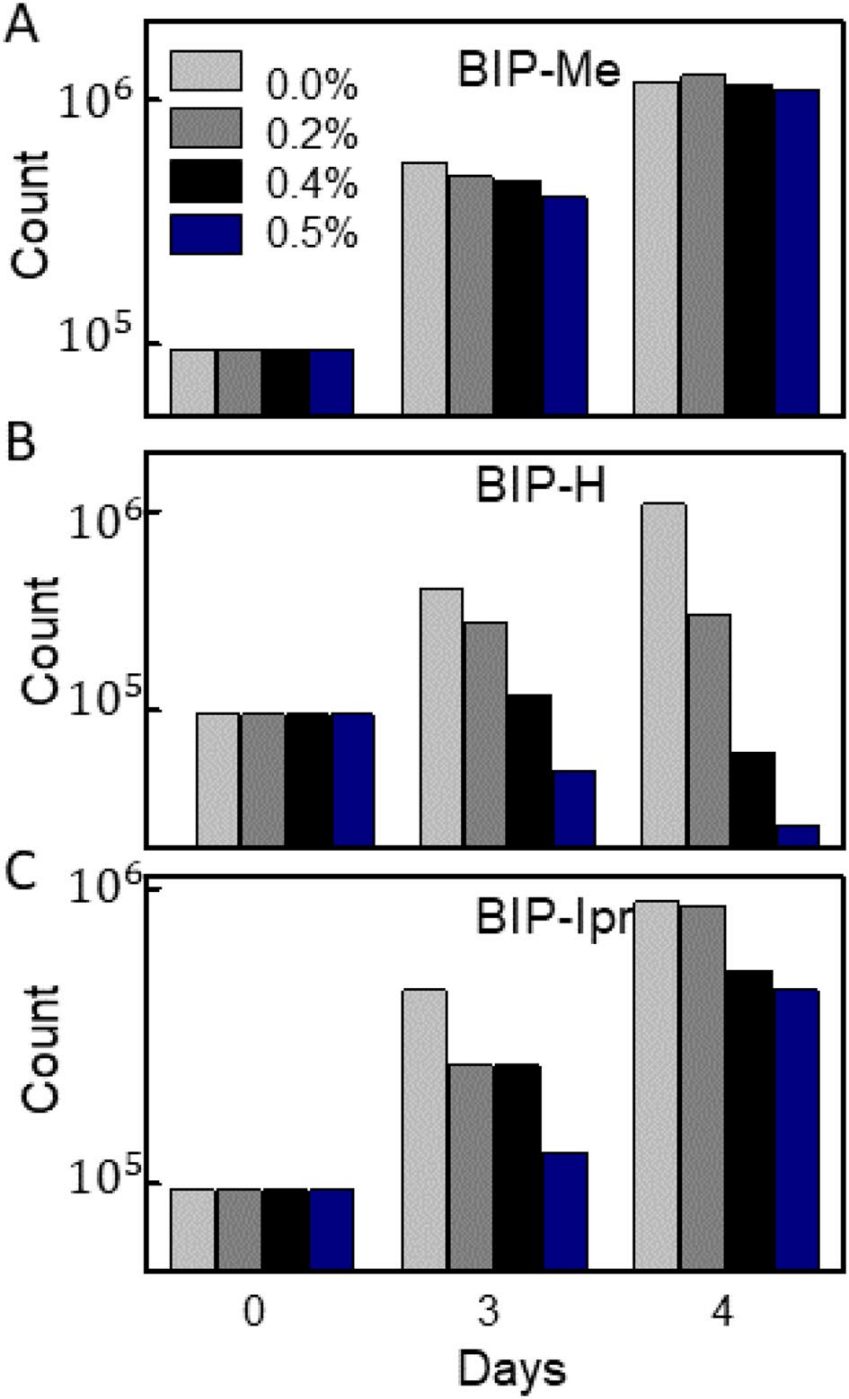
Cells counted over time show cytotoxicity results for (**A**) BIP-Me, (**B**) BIP-H and (**C**) BIP-IPr ligands taken over several days for different ligand concentrations by weight.

Apart from being able to controllably tune the shell sizes, the biocompatibility of the ligands is an additional parameter than needs evaluation. We examined the cell toxicity for the three ligands, BIP-Me, BIP-H, and BIP-IPr, shown in Fig. 6. C2C12 skeletal myoblasts (ATCC) were plated into 12 well-plates at a density of 10,000 cells/cm^2^, fed cell culture medium containing Dulbecco’s Modified Eagle Medium (DMEM), 10% fetal bovine serum (FBS), 2 mM glutamine, and 1 × 10^−4^ M nonessential amino acids, and cultured at 37 °C under 5% CO_2_. After 1 day, the media was replaced with 1 mL of new medium with the ligands added to the wells over a range of concentrations and cultured over time. Viability assays were performed with trypsin exclusion and counted on a using a hemocytometer. Figure 6A–C plot counts over Days 0–4 for cells with varying concentrations of ligands added to the cell culture medium. For every ligand concentration, six plates were used, and the average of each are plotted. BIP-Me and BIP-IPr appear to be tolerated well, even up to 0.5% ligand by weight, but BIP-H hinders cell growth above 0.2%. This moderately positive outcome is encouraging, indicating that when this versatile assembly process is performed with non-toxic QDs or metallic nanoparticles as the constituents.

## Conclusions

We have demonstrated that nanoshell self-assembly can be tuned in a liquid crystalline environment using non-mesogenic organic ligands to alter the enthalpy associated with the isotropic to nematic phase change. Differential scanning calorimetry confirms that shell size distributions are directly influenced by the non-mesogenic organic ligands. The influence of these ligands is predicated on their ability to iteratively depress the isotropic to nematic phase change temperature (T_IN_) of the liquid crystal host (5CB). This depression of T_IN_ on shell size is observed using both SEM and DLS to significantly influence the center of the size distribution of self-assembled structures. While further investigation of the host–guest interaction of liquid crystal facilitated self-assembly systems is being pursued, this work stands as one of the first examples of utilizing ligands to influence the thermodynamics of the host–guest interaction for directed assembly in a manner that controllably tunes the size of the assembled structures across the entire mesoscale. Further, the fact that the ligands are biocompatible to a significant extent open up possibilities of application in novel areas, such as in the field of tissue engineering, where the incorporation of nanomaterials is a relatively new effort. Nano-assembled capsules such as these could provide a platform for improved delivery of biological materials in vitro, for example, growth factors (GFs), which are proteins that direct cell growth and differentiation^35^. The standard approaches of either mixing the GFs into the extracellular matrix^36^ or allowing them to be absorbed on surfaces of interest^37^, suffer from a lack of control in terms of time of release, as well as inefficient retention. While some nano-assembled carriers^38–42^ have been devised, none have optimized the requirements of high loading efficiency, stability, and well-defined and predictable release profile. The size-tunable shells described here could resolve many of these issues, and combined with the success of earlier works^13^, may prove to be a model platform for in vitro cargo delivery in 3D tissue engineering applications.

## Abbreviations

LC: Liquid crystal
DLS: Dynamic light scattering
DSC: Differential scanning calorimetry
QD: Quantum dot
BIP: Bis imino pyridine
SL: Superlattice
NP: Nanoparticle
SEM: Scanning electron microscopy
ODA: Octadecylamine
